# Global Transmission, Spatial Segregation, and Recombination Determine the Long-Term Evolution and Epidemiology of Bovine Coronaviruses

**DOI:** 10.3390/v12050534

**Published:** 2020-05-13

**Authors:** Elias Salem, Vijaykrishna Dhanasekaran, Herve Cassard, Ben Hause, Sarah Maman, Gilles Meyer, Mariette F. Ducatez

**Affiliations:** 1 IHAP, Université de Toulouse, INRAE, ENVT, 31076 Toulouse, France; elias.salem@envt.fr (E.S.); herve.cassard@envt.fr (H.C.); gilles.meyer@envt.fr (G.M.); 2 Biomedicine Discovery Institute and Department of Microbiology, Monash University, Melbourne, VIC 3800, Australia; vijay.dhanasekaran@monash.edu; 3 Program in Emerging Infectious Diseases, Duke-NUS Medical School, Singapore 169857, Singapore; 4 Diagnostic Medicine/Pathobiology, College of Veterinary Medicine, Kansas State University, Manhattan Kansas, KS 66506-5802, USA; bhause@cambridgetechnologies.net; 5 SIGENAE, GenPhySE, Université de Toulouse, INRAE, INPT, ENVT, 31326 Castanet Tolosan, France; sarah.maman@inrae.fr

**Keywords:** bovine coronavirus, France, recombination, geographic clustering, molecular clock

## Abstract

Bovine coronavirus (BCoV) is widespread in cattle and wild ruminant populations throughout the world. The virus causes neonatal calf diarrhea and winter dysentery in adult cattle, as well as upper and lower respiratory tract infection in young cattle. We isolated and deep sequenced whole genomes of BCoV from calves with respiratory distress in the south–west of France and conducted a comparative genome analysis using globally collected BCoV sequences to provide insights into the genomic characteristics, evolutionary origins, and global diversity of BCoV. Molecular clock analyses allowed us to estimate that the BCoV ancestor emerged in the 1940s, and that two geographically distinct lineages diverged from the 1960s–1970s. A recombination event in the spike gene (breakpoint at nt 1100) may be at the origin of the genetic divergence sixty years ago. Little evidence of genetic mixing between the spatially segregated lineages was found, suggesting that BCoV genetic diversity is a result of a global transmission pathway that occurred during the last century. However, we found variation in evolution rates between the European and non-European lineages indicating differences in virus ecology.

## 1. Introduction

Bovine coronavirus (BCoV) is widespread in cattle and wild ruminant populations, resulting in neonatal calf diarrhea and winter dysentery in adult cattle, as well as upper and lower respiratory tract infection. BCoV is associated with the bovine respiratory disease complex (BRD), a leading cause of morbidity of young cattle worldwide with large economic costs due to mortality, treatment, and impeded growth performances, and pose a threat to public health as a result of antibiotic use. Having a multifactorial etiology, BRD is a complex disease triggered by the presence of one or several viruses and/or bacteria [1] favored by an altered state of the host immune system [2] and usually disturbed environmental factors [3] and several physical and biological stressors [2,4,5]. Viruses associated with BRD are capable of initiating a cascade of events such as immune suppression [2,6], respiratory epithelium damage, [7] and altering commensal microbiota and local biofilm [8,9,10], thus leading to complex secondary bacterial infections [8,10,11].

BCoV is a pneumoenteric virus frequently isolated from the fecal samples of diarrheic calves [12,13,14,15] and from upper and lower respiratory tract samples during BRD episodes worldwide [16,17,18,19]. While both forms of BCoV infection are widespread with high prevalence among cattle [12,13,15,20,21] and wild ruminants [22], the putative differences between strains isolated from enteric and respiratory tracts are still not clear, and it is unknown whether these differences are related to tissue tropism or simply to distinct times and locations of isolation. Some reports indeed suggest that all the BCoV are similar at genomic and antigenic levels [18,23], while others suggest that enteric and respiratory strains are genetically and antigenically different [24,25]. Zhang et al. highlighted molecular differences within a single host, with intra-host quasispecies and enteric strains more prone to genetic changes than their respiratory counterparts [26]. The global genetic and antigenic diversity of BCoV is also poorly understood. Using a short variable region of the BCoV genome and with sporadically collected samples from a few countries in Europe, Asia, and North America, it has been suggested that BCoV are segregated into European and American BCoV lineages [17,27,28,29], with periodic introductions of North American BCoV observed in Asian countries (for example in Japan during the 1990s [30]) highlighting global movement of BCoV likely through cattle trade.

Coronaviruses are enveloped particles with a large non-segmented, linear, polyadenylated, single-stranded positive sense RNA genome [31]. They belong to the family *Coronaviridae* in the order Nidovirales within the *Coronavirinae* subfamily [32]. Coronaviruses are classified into four genera [33]: *Alphacoronavirus*, *Betacoronavirus*, *Gammacoronavirus*, and *Deltacoronavirus*, and are known to infect birds and many mammals including humans [34]. BCoV are members of the *Betacoronavirus* genus within the *Coronaviridae* family, which includes several important human pathogens of zoonotic origin, such as the human coronavirus (HCoV) OC43, HKU1, MERS, SARS-CoV-1 [35], and SARS-CoV-2 [36,37], and mammalian viruses including equine coronaviruses (EqCoV) [38], canine respiratory coronaviruses (CRCoV) [39], swine hemagglutinating encephalomyelitis virus (HEV) [40], murine coronaviruses [32], and bat coronaviruses (BatCoV) HKU4, HKU5, and HKU9 [41,42,43,44]. BCoV are most closely related to members of the *Betacoronavirus1 (Embecovirus)* species PHEV, OC43, and EqCoV and share similarity in genome organization [32].

Coronaviruses pose a constant public health threat, due to their low replication fidelity and high genetic variability, making them prone to a constant changing pattern often leading to emerging diseases [45]. The intensive investigation that followed the emergence of SARS-CoV1 [46] and MERS-CoV [47] confirmed the zoonotic origins of those beta-coronaviruses [35,43,48,49,50], their efficient adaptability [51], and their potency to switch species [52]. SARS-CoV2 was also shown to have emerged from an animal reservoir and very successfully jumped into human causing the current pandemics [36,37]. Based on antigenic similarity it is thought that the human OC43 and canine respiratory coronaviruses emerged from BCoV [53,54].

The aim of the present study was to characterize BCoV from both upper and lower respiratory tracts of symptomatic animals in the south–west of France. We aimed to isolate the viruses, sequence their full genomes by deep sequencing, and analyze the obtained genetic data using phylogeny and time scale evolution models to better understand the virus evolution. While our starting point was French field BCoV, our study aims at a better understanding of BCoV genetic evolution at a global scale, looking both at the genetic characteristics of viruses and at the collection times in order to contribute to discussions on BCoV origin (in time and space) and global spread.

## 2. Materials and Methods

All methods were carried out in accordance with relevant guidelines and regulations. All experimental protocols were approved by the Ecole Nationale Vétérinaire de Toulouse and/or the Genotoul bioinformatics platform Toulouse Midi-Pyrénées.

### 2.1. Sample Collection and Processing

Deep nasal swabs (NS) and bronchoalveolar lavages (BALs) were collected from herds affected with respiratory disease in the south–west of France during 2014. The calves included in this study were not treated nor vaccinated prior to sampling. The inclusion criteria were: (i) calves from breeding farms of maximum five months of age; (ii) with acute signs of pneumonia during BRD episodes and clinical signs for maximum four days; (iii) minimum 60% of the herd with clinical signs; (iv) clinical signs: hyperthermia (>40 °C) or hyperthermia (>39.5 °C) with cough, snuffles, or tachypnea; (v) respiratory distress and abnormal lung sounds (rhonchi). Table 1 summarizes the details on sampled herds. Four calves were sampled from each of the affected herds, and samples were pooled separately by tissue type (NS or BAL). Swabs were inserted deep in the calf nostril (approximatively 20 cm deep) and rotated for 30 s against the nasal mucosae. Swabs were placed in PBS, vortexed, and kept on ice for no more than 2 h until being stored at −80 °C. BALs by bronchoscopy were performed under deep anesthesia: 10 mg/kg Diazepam and 10 mg/kg ketamine were injected intravenously, and a sterile flexible bronchoscope (Olympus, Tokyo, Japan) was passed through the pharynx and visually guided to the first bronchial division corresponding to the caudal segment of the right apical lung lobe. The lung lobe was then lavaged with 100 mL of sterile isotonic saline solution and the liquid was immediately aspirated after the infusion and refrigerated until further processing.

### 2.2. Sample Preparation and NGS Sequencing

Pools of both sample types were filtered by a syringe driven 0.45 µm filter, and the BALs were concentrated by centrifugation (100,000× *g*, 2 h, 4 °C). Three hundred microliters of PBS were added to the pellet and left at 4 °C overnight before suspension. All samples were then treated with a cocktail of nucleases for 1 h at 37 °C to eliminate the host nucleic acids (Exonuclease I (20U, Thermo Fischer, Waltham, MA, USA), Benzonase (25U, Merck chemical, Darmstadt, Germany), Turbo DNAse (2U, Thermo Fisher Scientific, Waltham, MA, USA), and RNase I (10U, Thermo Fischer, Waltham, MA, USA). Viral nucleic acids were purified using the QIAamp MinElute virus spin kit (Qiagen, Hilden, Germany). Sequence-independent, single primer amplification was carried out as described by Liais et al. [55]. Briefly, reverse transcription was performed using tagged random hexamers, first and second strands were then randomly amplified by PCR. PCR products were purified and libraries processed using the Truseq nano kit (Illumina, San Diego, CA, USA) for next-generation sequencing with Illumina Miseq v2 platform (San Diego, CA, USA) generating paired 250 bp reads. Bioinformatics analysis was carried out using an in-house bioinformatics pipeline. Briefly, sequence reads from each library were filtered, demultiplexed, sorted, and pair-end reads merged. Viral composition and relative abundance were determined using GAAS [56], the metagenomes were compared to NCBI RefSeq virus database using Blastx. BCoV reads were also mapped against the Mebus BCoV genome (accession U00735.2) using the Burrows–Wheeler Alignment Tool. Reads of BCoV were as well assembled using CLC Genomics Workbench (Qiagen, Hilden, Germany). Specific primers were designed based on the extracted reads for RT PCR to fill sequence gaps between the contigs and the produced amplicons were sequenced on a 3130XL Applied Biosystems capillary sequencer (Applied Biosystems, Foster City, CA, USA).

### 2.3. Screening for Bovine Respiratory Pathogens

Samples were screened using the VetMAX Screening pack–Ruminant Respiratory Pathogens real-time PCR kit (Thermo Fischer, Waltham, MA, USA) according to the manufacturer instructions for the detection of *Mycoplasma bovis*, *Histophilus somni*, *Pasteurella multocida*, *Mannheimia haemolytica*, bovine coronavirus, bovine respiratory syncytial virus (BRSV), and bovine parainfluenza-3 virus (BPI-3).

### 2.4. BCoV Isolation

Virus isolation was attempted in Human Rectal Tumor-18G (HRT-18G) cells as follows: samples filtered by a syringe driven 0.22 µM filter were propagated on HRT-18G (ATCC, Manassas, VA, USA) cells in the presence of TPCK trypsin (1 µg/mL, Thermo Fisher Scientific, Waltham, MA, USA) in optiMEM Reduced Serum Media (Thermo Fisher Scientific, Waltham, MA, USA) supplemented with Penicillin (100 units/mL, Waltham, MA, USA), Streptomycin (10 mg/mL; Life technologies Carlsbad, CA, USA), Ciprofloxacin (10 µg/mL, Sigma–Aldrich, St Louis, MO, USA), Amphotericin B (2.5 µg/mL, Sigma–Aldrich, St Louis, MO, USA), and BM-cyclin (15 µg/mL, Sigma–Aldrich, St Louis, MO, USA). The cells were incubated for four days at 37 °C before pooling and filtration of the supernatant.

### 2.5. Virus sequence Datasets and Phylogenetic Analysis

Viruses sequenced in this study were combined with BCoV nucleotide sequence data available in GenBank, along with their sampling dates, host, and sample type (respiratory or fecal) (Supplementary Tables S1 and S2). We focused on the spike (S) and nucleocapsid (N) genes as the large majority of the available BCoV sequence data was on S and N genes. Multiple sequence alignments were generated with Clustal Omega [57,58] and manually optimized with BioEdit v7.1 [59]. Maximum likelihood (ML) trees were constructed in MEGA v6 [60] using the best-fit nucleotide substitution models: Tamura–Nei model with gamma-distributed rate variation (TN93+G) for N gene and the general time reversible model with gamma-distributed rate variation and a proportion of invariant sites (GTR+G+I) for the S gene. The quality of nucleotide sequences along with their temporal signal were assessed using a regression of the root-to-tip distances of the ML tree and virus sampling dates in TempEst v1.5 [61]. Samples that deviated from the regression were assumed to either be erroneous or incorrectly labelled and were removed from subsequent analysis.

### 2.6. Recombination Detection

Recombination detection was conducted for the N and S gene datasets using the Genetic Algorithm of Recombination Detection (GARD) method [62] in the Datamonkey server of HyPhy v2 [62,63]. Datasets of recombination-free sequences were constructed by dividing the original alignments at the detected breakpoints and were used in all subsequent analyses.

### 2.7. Molecular Dating

Nucleotide substitution rates and the evolutionary time scale of divergence of BCoV lineages were estimated using the year of virus sampling used as tip-calibrations in a relaxed molecular clock method under the Bayesian Markov chain Monte Carlo (MCMC) framework in BEAST v1.7.1 [64], implemented on a Galaxy workbench (http://galaxy-workbench.toulouse.inra.fr/). Molecular clock branch rates were derived from a log-normal distribution [65]. The Hasegawa–Kishino–Yano (HKY) nucleotide substitution model was specified separately for codon positions 1 + 2 and 3 and used a constant population size coalescent model. Analysis were run for 2 × 10^8^ generations, sampling every 10,000 generations. Defaults were used for all priors expect for the clock rate for which a uniform prior distribution was specified with an initial value of 10^−4^ substitutions/nucleotide/year, based on previous estimates of coronavirus substitution rates [66]. Convergence of each of the parameters with a minimum effective sample size of 500, following a burning of 10%, was assessed using Tracer [64]. Maximum clade credible trees with the mean time of the most recent common ancestor (tMRCA) and their 95% highest posterior density (HPDs) were generated using the TreeAnnotator program included in the BEAST package and visualized in FigTree v1.4.3.

### 2.8. Selective Pressure Analysis

Selection pressure acting on the S gene were investigated by estimating the ratio between the mean number of non-synonymous (dN) and synonymous (dS) nucleotide substitution site (dN/dS) using the Single Likelihood Ancestor Counting (SLAC), Fixed Effects likelihood (FEL) [67,68] and the Mixed Effects Model of Evolution (MEME) method implemented in the datamonkey server of HyPhy v2 [63].

## 3. Results

### 3.1. Genomic Surveillance of BCoV in France

Upper (NS) and lower (BAL) respiratory tract samples were collected from 16 calves exhibiting acute respiratory disease during 2014 in the south–west of France (two respiratory samples per calf, four samples per pool; Table 1). Screening for other respiratory pathogens using real-time PCR resulted in the identification of co-infecting agents (Table 1), including *P. multocida*, *M. haemolytica*, *M. bovis*, and *H. somni*. All samples had at least one co-infecting pathogen, with *P. multocida* in 3/4 calves, followed by *M. haemolytica*, BRSV, *M. bovis*, BPI-3, and *H. somni* (Table 1). This suggests that the respiratory clinical signs were not due to BCoV alone but to co-infecting pathogens. A genetic focus was however made on BCoV in the present study. A greater proportion of BCoV were detected in NS samples (with lower Ct values) compared to BAL in three out of four pools studied.

Nucleic acids extracted from purified samples were subjected to NGS sequencing, focusing on BCoV genetic material arising separately from the upper and lower respiratory samples of infected cattle. In total, five complete and two partial BCoV genomes were generated (accession numbers MG757138-44), from which the nucleocapsid (N) gene and spike (S) gene sequences were extracted and utilized for subsequent evolutionary analysis (Supplementary Tables S1 and S2). Attempts of virus isolation in HRT-18G cells resulted in the successful isolation of one strain, BCoV/France/ICSA17_LBA/2014, collected from the BAL sample of infected calf in 2014.

### 3.2. Recombination of BCoV

Since coronavirus genomes are known to undergo recombination, we inferred the occurrence of recombinant breakpoints along the N and S gene alignments of BCoV collected since the 1960s, and found one recombination breakpoint in the S gene at nucleotide 1101 with significant differences in evolutionary histories (KH test) between the two loci at nucleotide positions 1–1100 and 1101–4089, whereas no breakpoints were detected in the N gene. These results suggest that a single phylogenetic tree may not accurately describe the evolutionary history of S gene and data were therefore partitioned for subsequent phylogenetic analysis.

### 3.3. Phylogenetic Relationships and Spatio-Temporal Evolution of BCoV

**Figure 1:**
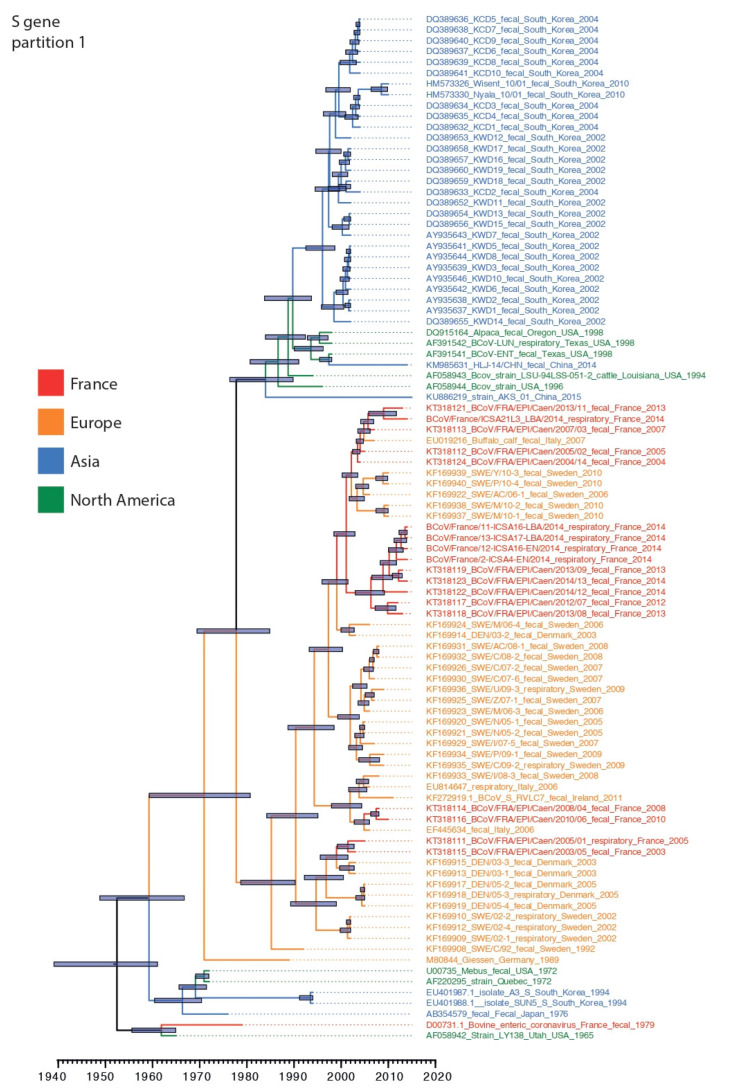
Dated phylogeny of BCoV S gene partition-1 (nt 1–1100). Tree generated using the relaxed clock model and a constant coalescent tree prior. Nodes correspond to mean tMRCAs. 95% highest posterior density (HPD) are indicated on nodes with grey boxes. French sequences (including those generated in the present study), European, Asian, and North American sequences are in red, orange, blue, and green fonts, respectively.

**Figure 2:**
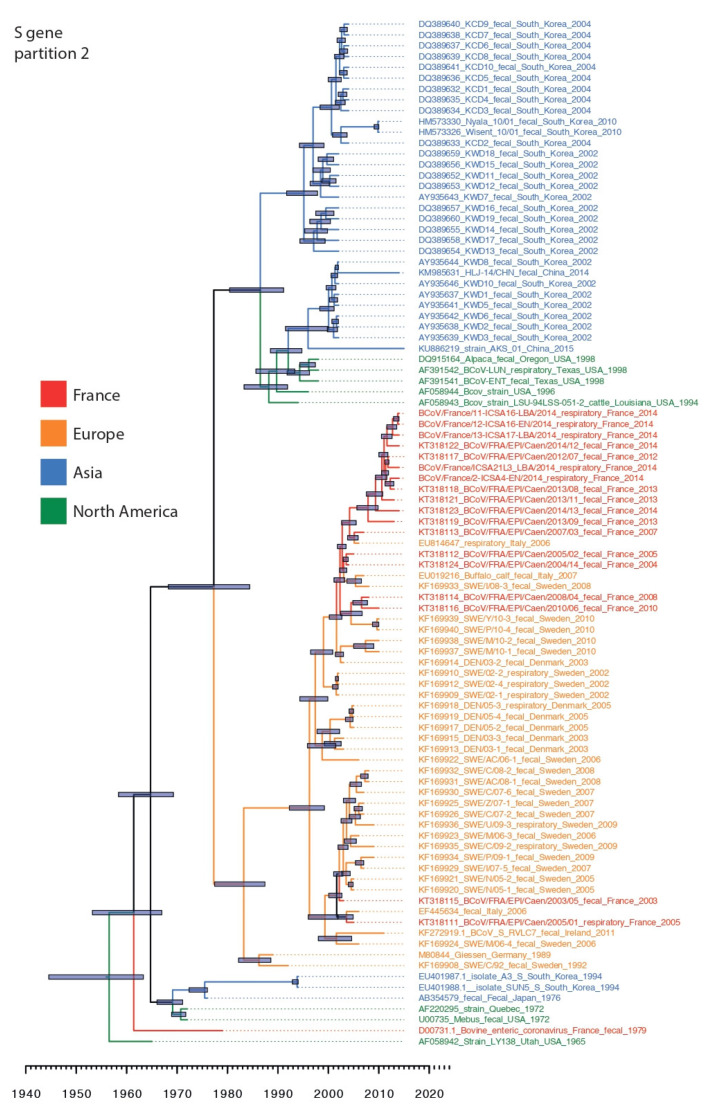
Dated phylogeny of BCoV S gene partition-2 (nt 1101–4089). Tree generated using the relaxed clock model and a constant coalescent tree prior. Nodes correspond to mean tMRCAs. 95% HPD are indicated on nodes with grey boxes. French sequences (including those generated in the present study), European, Asian, and North American sequences are in red, orange, blue, and green fonts, respectively.

Phylogenetic analysis was conducted using 95 BCoV S gene sequences including seven from the present study, and 54 BCoV N gene sequences including five newly generated sequences. Our sequence dataset was predominantly restricted to sequences originating from North America, Asia and Europe highlighting the paucity of BCoV surveillance and the lack of well curated sequence datasets from around the globe. S gene alignments were partitioned at the detected recombination breakpoint to create S gene datasets ranging from nucleotide positions 1–1100 and 1101–4089. In both data partitions, BCoV circulating since the 1990s formed two broad spatially segregated phylogenetic groups in Asia and Europe, respectively, derived from Mebus/1972-like strain collected in North America (Figure 1 and Figure 2). There were several topological differences between the S gene data partitions that occurred both during the emergence of lineages in Europe and Asia, but we also identified topological differences within the Asian and European clade suggesting continued recombination among co-circulating lineages following spatial segregation (Figure 1 and Figure 2).

The Asian lineage of BCoV was predominated by strains collected in South Korea during 2002–2010 and both S gene partition phylogenies suggest that these strains were derived from strains similar to those reported from North America during 1994–1998. Estimation of tMRCA of the South Korean lineages showed that the mean tMRCA of S gene loci-1 was significantly more recent during 1995 (mean tMRCA: 1995; highest posterior density (HPD): 1992.5–1998.7) than in data partition-2 which constituted two distinct lineages that diverged during 1986 (mean tMRCA: 1986; HPD: 1980–1991), however the tMRCAs of the two co-circulating lineages overlapped with the tMRCA of partition-1 highlighting the co-circulation of multiple lineages in South Korea (Table 2). In addition, two strains collected recently from cattle in China exhibited differences in origin between the S data partitions: their first 1000 nucleotides appear to be derived independently from strains similar to those collected in North America during 1994–1998, whereas the second but longer partition was derived directly from South Korean specimens collected during 2002–2010. These results suggest historical movement of BCoV between the North America and Eurasia landmasses, as well as between Asian countries.

The European lineage (EU-lineage) was represented by BCoV collected from several countries in Europe since the 1990s, including France, Denmark, Germany, Italy, and Sweden; however, excluding France, most BCoV sequence data in Europe derives from 2002–2010. There is also evidence that the EU-lineage evolved through recombination as the ancestral relationship of these lineages to M80844/Geissen-Germany/1989 differed between the two loci (Figure 1 and Figure 2), indicating that the recombination event (breakpoint at nt position 1100) may be at the origin of the distinct Asian and European lineages.

BCoV sequences collected from calves in France between 2013–2014 revealed a greater genetic heterogeneity in S gene loci-1 (1–1000 nt) with evidence of co-circulation of two lineages that diverged as early as 2000 (mean tMRCA: Aug 2000; 95% HPD: 1998.5–2009.8; Figure 1), whereas loci-2 exhibited fewer differences between contemporary viruses and revealed a mean divergence of approximately four years (mean tMRCA: Mar 2009; 95% HPD: 2007.5–2010.8). When looking specifically at the south-western French BCoV sequences, BCoV/France/ICSA21L3/2014 differed from its counterparts on locus 1 (being closely related to a recent north-western French sequence, BCoV/France/EPI-Caen/2013) but clustered with its south-western counterparts for the locus 2 (Figure 2). These results highlight a complex evolutionary history that is determined by migration and pervasive recombination.

Our N gene dataset comprised lower sampling outside of France (Table S2). All N gene sequences of BCoV strains from France, isolated since 2003, formed a monophyletic group, with the mean tMRCA of the contemporary BCoV (2013–2014) isolates estimated during 2004 (95% HPD: 1999.2–2009.2), indicating a result that overlaps with the tMRCA of both S gene partitions.

We estimated the evolutionary rate for the S and N gene datasets using the relaxed clock model, and found a relatively lower rates of nucleotide substitution in BCoV datasets, ranging from a mean rate of 0.81 × 10^−3^ (95% HPD: 0.5–0.8 × 10^−3^) for S gene data partition-1, 1.0 × 10^−3^ (HPD: 0.7–1.3 × 10^−3^) for S gene data partition-2 and a lower but with wide-confidence intervals for the N gene dataset (mean rate: 0.2 × 10^−3^; 95% HPD: 0.1–0.4 × 10^−3^). These estimates were similar to those estimated for other coronaviruses, for example the mean nucleotide substitution rate of MERS was estimated at 1.1 × 10^−3^ (95% CI: 0.8–1.4 × 10^−3^) [69]. Estimates of the coefficient of variance of the relaxed clock model ranged from a mean of 0.61 for the S gene partition-1, to 0.7 for S partition-2 and the N gene datasets, and HPD intervals of all datasets were lower than 1, indicating that there was significant variation in nucleotide substitution rates along the BCoV branches, and also suggesting that the relaxed clock model was more appropriate than using a strict clock model.

The tMRCA of globally sampled BCoV for the N and S genes was estimated during 1940 and 1964, suggesting the common ancestor for known BCoV was about 51 to 75 years prior to the last included sequence date (2015). Whereas the most common recent ancestor of the EU-lineage was 1978–1981 (Figure 1 and Figure 2). The recombination event that likely led to the two main lineages with clear geographic distinction (US/Asia and European lineages) co-circulating today may thus have occurred in the 1960s–1970s.

### 3.4. Selection Pressure

The selection analysis for the S gene was conducted using two partitions. The average ratio between the mean number of non-synonymous (dN) and synonymous (dS) nucleotide substitution sites (dN/dS) was estimated to be 0.310 for the S gene, with 28 positively selected amino acids (from SLAC, FEL and MEME; Table 3). Amino acids 35, 113, 115, 447, 499, 501, 525, 716, 893, and 1239 were detected as positively selected in at least two of the three used models (Table 3).

## 4. Discussion

### 4.1. Prevalence of BCoV in Europe

Bovine coronavirus is considered widespread in Europe and worldwide in both its enteric and respiratory forms. Furthermore, BCoV is considered a main pathogen associated with pneumonia in feedlots in North America [15,16,70,71]. Although BCoV is no less a very common pathogen in Europe, limited data is available on its epidemiology. Norwegian studies have revealed a high seroprevalence in cattle with 39.3% and 80.7% in calves and herds, respectively, in 2009, and 72.2% in herds tested in 2016 [21,72]. In Italy, four respiratory disease outbreaks during 2006 were associated with BCoV without concurrent detection of other known respiratory pathogen but with the detection of BCoV in the intestines in two of the four outbreaks [19]. In Belgium, seroconversion for BCoV was detected in 30% of veal calves herds [73]. In a retrospective study in Ireland (2008–2012), BCoV was the most frequently detected virus in cattle nasal swabs from respiratory disease outbreaks [74]. In France, BCoV was detected in 17%–63% of respiratory samples collected in two independent studies [75]. Taken together, literature on BCoV in Europe is limited but highlights the high circulation of the pathogen at the continental scale.

### 4.2. No obvious Link between Virus Tropism and Genetic Markers

It is so far unknown whether a unique or distinct BCoV are responsible for respiratory and enteric infections [18,23,25,76]. As recently observed in Austria and Slovakia [23], we did not succeed in identifying genetic determinants of tissue tropism, nor between enteric and respiratory BCoV strains (based on published sequences), nor between strains present in the upper and lower respiratory tracts (based on the sequences generated within the framework of the present study). In the absence of viral genetic determinants of tissue tropism, the immune status of the animals at the time of the BCoV infection, infectious dose, and route of inoculation (oro-fecal versus aerosol) may play a role in determining sites of infection [77]. In addition, the animals sampled in the present study suffered from respiratory disease, but as both BCoV and co-infecting pathogens were detected, a direct link between BCoV and respiratory signs cannot be made. Further studies are clearly warranted to understand the mechanisms behind the differential tissue tropism of BCoV.

### 4.3. Little Exchanges of BCoV between Europe and the Other Continents

Among available BCoV complete genomes, a minority are of European origins. In this study we therefore characterized respiratory strains of BCoV, from both upper and lower respiratory tracts, at the genetic level. Despite recent efforts, there are notable gaps in our understanding of bovine coronavirus dynamics and diversity. Our results show that the French strains described here cluster with all other Europeans strains, thus indicating a homogeneous evolution of BCoV in Europe, whereas a greater mixing was observed between isolates collected in North America and Europe. This pattern of geographical and temporal distinction between strains was previously described for BRSV, for which distinct phylogenic groups with continent-specific strains were identified [78,79,80]. Similarly, BVDV also harbors a geographic clustering [81]. This peculiar evolutionary process could be explained by the relative isolation of Europe as far as cattle traffic is concerned. According to FAOstat [82], cattle traffic involving Europe is mainly unidirectional with export of live animals from France (for the most part) to other continents with a negligible importation activity. On the other hand, the import/export activity between North America and East Asia is much more fluid. In addition, Kin et al. recently highlighted this important trade constraint on BCoV spread at a global scale [28]. It is however unclear why/how BCoV, BRSV, or BVDV would spread differently than other bovine RNA viruses. Influenza D viruses (IDV), which primarily infects bovine hosts, seem for example to have emerged recently but genetically and anti-genetically similar viruses circulate in Europe and North America [29,83,84,85,86]. One may therefore hypothesize that different routes of transmission may be used for the different pathogens. In addition, human coronaviruses (OC43 and 229E) were shown to induce short-term immunity [87]. If BCoV led to similar responses in cattle, herds with animals of multiple ages (as seen more often in France than in North America) might face re-infections with BCoV and might maintain virus over long periods of time, which would impact the virus evolution.

Recombination likely drove the different evolutionary process taken by the Asian and North American strains and the Europeans strains; in the first partition of the S gene dataset, the strains M80844 Giessen/1989 clustered along with French strain D00731/1979, US strain AF058942 LY138 Utah/1965, South Koreans EU401988 SUN5/1994 and EU401987 A3/1994, and North Americans AF220295 Quebec/1972 and U00735 Mebus/1972, suggesting a common ancestor of this cluster. Whereas, in the partition-2 of the S dataset, we noticed a change in the phylogenic scheme: the M80844 Giessen/1989 clustered with all Europeans strains, while the more recent North Korean (year 2002) and recent Chinese KM985631 HLJ/T4/2014 and KU886219 AKS/01/2015 clustered with the US strains of the 1990s, suggesting a common origin. The old BCoV strains (pre 1994) seemed to have the same origin irrespective of their geographic origin until an event of recombination led the European strains to evolve separately.

### 4.4. Evolution of BCOV

Using the different models (constant population under strict and relaxed clock for all the strains and the constant population and BSP under relaxed clock for the European strains), the mean substitution rates calculated for the spike genes ranged between 3.9 and 9.8 × 10^−4^ nucleotide substitution per site per year, which is a very similar range as previously published (2.1-10.5 × 10^−4^ [88,89]). The slower evolution of N than of S gene is also in line with previous reports from the literature [89]. Substitution rates were in the same range for other coronaviruses with 0.6 × 10^−3^, 0.7 ± 2 × 10^−3^ for HCoV-OC43 and TGEV, respectively [90,91]. We observed a slightly faster evolution for European BCoV than for the whole dataset. An important bias in our analyses comes from the lack of sequence data and a timing difference between European and American BCoV, such that most North American specimens were at least ten years older than European strains. In addition, the production systems (different numbers of animals per farms, feedlots in North America but not in Europe), the virus prevalence, and the vaccine coverages are likely different, therefore the selection pressures must be different. Our selection pressure calculations unfortunately did not allow for highlighting any difference in selection pressure between the continents. The 10 positively selected amino acids detected here (Table 3) were spread out over the whole spike protein and could not be linked to a biological function due to the limited research so far carried out on BCoV; no spike structure has so far been resolved (comparison is only possible with the SARS spike), and no clear antigenic sites have been detected to the best of our knowledge. Further studies are thus warranted to understand the role of these positions in BCoV evolution.

As far as the tMRCA is concerned, our analysis with the new sequence data confirmed the values previously estimated: 1978 (95% CI: 1910–1963) or 1944 (95% CI: 1910–1963) [88,89]. Here again, more sequences available throughout the last few decades would allow for an increased precision in the tMRCA. We showed here for the first time that a recombination event may be at the origin of the viruses that evolved separately in Europe on the one side and in America/Asia on the other side. This event likely occurred in the 1960s–1970s, when cattle exchanges between Europe and the other continents was already very limited.

## 5. Conclusions

Taken together, our results showed that the BCoV ancestor emerged in the 1940s, and that two geographically distinct lineages diverged from the 1960s–1970s with no genetic mixing detected thus far. Further studies are warranted to understand the mechanisms behind BCoV tissue tropism. Improved surveillance and sequencing are needed to precisely estimate the evolutionary and epidemiological parameters of BCoV circulation and to identify the recombinations in genomic evolution.

## Figures and Tables

**Table 1:** Presence of co-infecting pathogens in bronchoalveolar lavages (BAL) or nasal swabs (NS) pools as determined by real time using the vetmax screening kit.

Sample		Ct Value ^2^						
Id	Type ^1^	*P. multocida*	*M. haemolytica*	*M. bovis*	*H. somni*	BCoV	RSV	PI-3
ICSA-4	BAL	26.75				30.17	22.74	
ICSA-16	BAL				39.01	24.19		
ICSA-17	BAL	27.04				38.9		
ICSA 21	BAL	29.32	27.31	29.27		38.61		
ICSA-4	NS	32.1				27.9	29.4	32.61
ICSA-16	NS					18.67		
ICSA-17	NS	28.9						
ICSA 21	NS	24.4	26.7			34.93		

^1^ BAL, bronchoalveolar lavage and NS, nasal swabs. ^2^ Ct values >40 are not shown. *P.*: *Pasteurella*; *M.*: *Mycoplasma*; *H.*: *Histophilus*; BCoV: bovine coronavirus; RSV: bovine respiratory syncytial virus; PI-3: bovine parainfluenza-3 virus.

**Table 2:** Evolution rates and TMRCA for N and S gene partitions.

Data	Gene	Demographic Model ^a^	Mean nt Sub Rate (95% HPD) × 10^−3 b^	Mean tMRCA (95% HPD) in Years before 2015
Global	S partition 1	Constant	0.8 (0.5–1)	63.5 (53.9–75.9)
	S partition 2	Constant	0.1 (0.7–1)	60.94 (51.7–70.4)
	N	Constant	0.2 (0.1–0.4)	77.9 (46.2–123.2)
Europe	S partition 1	Constant	0.7 (0.5–0.9)	41.6 (37.5–53.5)
		Bayesian skyline	0.7 (0.5–0.9)	41.9 (36.4–52.2)
	S partition 2	Constant	0.8 (0.6–0.9)	39. 7 (35.3–54.4)
		Bayesian skyline	0.8 (0.6–1.0)	37.5 (35.0–47.9)
	N	Bayesian skyline	0.5 (0.3–0.7)	35.8 (35.0–45.4)
		BSP	0.5 (0.3–0.7)	35.6 (35.0–46.0

^a^ Bayesian skyline model is a flexible coalescent prior that allows variation in population size over time. ^b^ estimated using the uncorrelated log-normal relaxed clock model and a constant population size model.

**Table 3:** Amino acid sites detected to be under positive selection (with *p*-values < 0.1) through either of the selection methods (SLAC, FEL, and MEME).

Codon	SLAC *p*-Value	FEL *p*-Value	MEME *p*-Value
35	0.0600	0.0700	**0.0130**
113	**0.0340**	**0.0220**	0.0700
115	0.0670	**0.0210**	#N/A
155	#N/A	#N/A	0.0500
179	#N/A	#N/A	0.0680
257	#N/A	0.0820	#N/A
304	#N/A	#N/A	**0.0160**
447	**0.0260**	**0.0260**	0.0660
484	#N/A	#N/A	0.0980
499	**0.0230**	**0.0090**	#N/A
501	**0.0010**	**0.0040**	**0.0060**
509	#N/A	**0.0410**	#N/A
525	**0.0220**	**0.0280**	0.0910
543	#N/A	#N/A	0.0760
689	#N/A	#N/A	**0.0380**
716	0.0590	**0.0490**	#N/A
744	#N/A	#N/A	**0.0060**
767	#N/A	#N/A	**0.0000**
769	#N/A	#N/A	0.0940
892	#N/A	#N/A	0.0710
893	#N/A	**0.0410**	**0.0010**
1085	#N/A	#N/A	**0.0030**
1188	#N/A	#N/A	0.0040
1206	#N/A	**0.0450**	#N/A
1237	#N/A	#N/A	**0.0380**
1239	#N/A	**0.0110**	0.0960
1269	#N/A	#N/A	**0.0000**
1362	#N/A	**0.0150**	#N/A

Sites with *p*-values < 0.05 are shown in bold. Codons positively selected as shown by at least two of the three selection methods are underlined. #N/A: no positive selection detected.

## Supplementary Materials

The following are available online at https://www.mdpi.com/1999-4915/12/5/534/s1, Table S1: Details of the strains used for *S* gene sequences analyses, Table S2: Details of the strains used for *N* gene sequences analyses.
