# Supplementary material for: Global Transmission, Spatial Segregation, and Recombination Determine the Long-Term Evolution and Epidemiology of Bovine Coronaviruses
Source: Viruses. 2020 May 13;12(5):534. doi: 10.3390/v12050534 (PMC7290379; doi:10.3390/v12050534)
Supplement: Supplementary file 1 [file viruses-12-00534-s001.pdf]

viruses

MDPI

Type of the Paper (Article)

Global transmission, spatial segregation and recombination determine the long-term evolution and epidemiology of Bovine Coronaviruses

E. Salem[1], D. Vijaykrishna[2,3], H. Cassard[1], B. Hause[4], S. Maman[5], G. Meyer[1], M.F. Ducatez[1]*

[1] IHAP, Université de Toulouse, INRAE, ENVT, Toulouse, France
[2] Biomedicine Discovery Institute and Department of Microbiology, Monash University, Melbourne, Victoria 3800, Australia
[3] Program in Emerging Infectious Diseases, Duke-NUS Medical School, Singapore
[4] Diagnostic Medicine/Pathobiology, College of Veterinary Medicine, Kansas State University, Manhattan Kansas KS 66506-5802, USA
[5] SIGENAE, GenPhySE, Université de Toulouse, INRAE, INPT, ENVT, Castanet Tolosan, France.

* Correspondence: mariette.ducatez@envt.fr

Supplementary Materials:

Supplementary Table 1. Details of the strains used for S gene sequences analyses

Accession number	Strain/isolate	Host species	Type of sample	Country	Sampling year
KU886219	AKS 1	bovine	-	China	2015
DQ389658	KWD17	bovine	enteric	South Korea	2002
DQ389656	KWD15	bovine	enteric	South Korea	2002
DQ389655	KWD14	bovine	enteric	South Korea	2002
DQ389653	KWD12	bovine	enteric	South Korea	2002
HM573330	Nyala 10.01	Nyala	enteric	South Korea	2010
HM573326	Wisent 10.01	Wisent	enteric	South Korea	2010
DQ389633	KCD2	bovine	enteric	South Korea	2004
DQ389654	KWD13	bovine	enteric	South Korea	2002
DQ389634	KCD3	bovine	enteric	South Korea	2004
DQ389635	KCD4	bovine	enteric	South Korea	2004
DQ389632	KCD1	bovine	enteric	South Korea	2004
DQ389636	KCD5	bovine	enteric	South Korea	2004
DQ389639	KCD8	bovine	enteric	South Korea	2004
DQ389641	KCD10	bovine	enteric	South Korea	2004
DQ389637	KCD6	bovine	enteric	South Korea	2004
DQ389638	KCD7	bovine	enteric	South Korea	2004
DQ389640	KCD9	bovine	enteric	South Korea	2004
DQ389659	KWD18	bovine	enteric	South Korea	2002
AY935643	KWD7	bovine	enteric	South Korea	2002
DQ389652	KWD11	bovine	enteric	South Korea	2002
DQ389657	KWD16	bovine	enteric	South Korea	2002
DQ389660	KWD19	bovine	enteric	South Korea	2002
KM985631	HLJ-14/CHN	bovine	enteric	China	2014
AY935641	KWD5	bovine	enteric	South Korea	2002
AY935646	KWD10	bovine	enteric	South Korea	2002
AY935637	KWD1	bovine	enteric	South Korea	2002
AY935642	KWD6	bovine	enteric	South Korea	2002
AY935638	KWD2	bovine	enteric	South Korea	2002
AY935644	KWD8	bovine	enteric	South Korea	2002
AY935639	KWD3	bovine	enteric	South Korea	2002
AB354579	kakegawa	bovine	enteric	Japan	1976
EU401988.1	SUN5	bovine	enteric	South Korea	1994
EU401987.1	A3	bovine	enteric	South Korea	1994
MG757144	BCoV/France/13-ICSA17-LBA/2014	bovine	respiratory	France	2014
MG757140	BCoV/France/11-ICSA16-LBA/2014	bovine	respiratory	France	2014
MG757139	BCoV/France/12-ICSA16-EN/2014	bovine	respiratory	France	2014

MG757138	BCoV/France/ICSA21L3/LBA/2014	bovine	respiratory	France	2014
MG757143	BCoV/France/2-ICSA4-EN/2014	bovine	respiratory	France	2014
KF169922	SWE/AC/06-1	bovine	enteric	Sweden	2006
KF169924	SWE/M/06-4	bovine	enteric	Sweden	2006
KF169940	SWE/P/10-4	bovine	enteric	Sweden	2010
KF169939	SWE/Y/10-3	bovine	enteric	Sweden	2010
KF169938	SWE/M/10-2	bovine	enteric	Sweden	2010
KF169937	SWE/M/10-1	bovine	enteric	Sweden	2010
KT318119	BCoV/FRA/EPI/Caen/2013/09	bovine	enteric	France	2013
KT318123	BCoV/FRA/EPI/Caen/2014/13	bovine	enteric	France	2014
KT318121	BCoV/FRA/EPI/Caen/2013/11	bovine	enteric	France	2013
KT318122	BCoV/FRA/EPI/Caen/2014/12	bovine	enteric	France	2014
KT318118	BCoV/FRA/EPI/Caen/2013/08	bovine	enteric	France	2013
KT318117	BCoV/FRA/EPI/Caen/2012/07	bovine	enteric	France	2012
KF169914	DEN/03-2	bovine	enteric	Denmark	2003
EU019216	Buffalo	Buffalo	enteric	Italy	2007
KT318112	BCoV/FRA/EPI/Caen/2005/02	bovine	enteric	France	2005
KT318124	BCoV/FRA/EPI/Caen/2004/14	bovine	enteric	France	2004
KT318113	BCoV/FRA/EPI/Caen/2007/03	bovine	enteric	France	2007
KF169933	SWE/I/08-3	bovine	enteric	Sweden	2008
EU814647	438/06-TN	bovine	respiratory	Italy	2006
KT318114	BCoV/FRA/EPI/Caen/2008/04	bovine	enteric	France	2008
KT318116	BCoV/FRA/EPI/Caen/2010/06	bovine	enteric	France	2010
KF169909	SWE/02-1	bovine	respiratory	Sweden	2002
KF169910	SWE/02-2	bovine	respiratory	Sweden	2002
KF169912	SWE/02-4	bovine	respiratory	Sweden	2002
KF169935	SWE/C/09-2	bovine	respiratory	Sweden	2009
KF169929	SWE/I/07-5	bovine	enteric	Sweden	2007
KF169934	SWE/P/09-1	bovine	enteric	Sweden	2009
KF169921	SWE/N/05-2	bovine	enteric	Sweden	2005
KF169920	SWE/N/05-1	bovine	enteric	Sweden	2005
KF169923	SWE/M/06-3	bovine	enteric	Sweden	2006
KF169936	SWE/U/09-3	bovine	respiratory	Sweden	2009
KF169930	SWE/C/07-6	bovine	enteric	Sweden	2007
KF169925	SWE/Z/07-1	bovine	enteric	Sweden	2007
KF169932	SWE/C/08-2	bovine	enteric	Sweden	2008
KF169931	SWE/AC/08-1	bovine	enteric	Sweden	2008
KF169926	SWE/C/07-2	bovine	enteric	Sweden	2007
EF445634	339/06	bovine	enteric	Italy	2006
KF169917	DEN/05-2	bovine	enteric	Denmark	2005
KF169919	DEN/05-4	bovine	enteric	Denmark	2005

KF169918	DEN/05-3	bovine	respiratory	Denmark	2005
KT318111	BCoV/FRA/EPI/Caen/2005/01	bovine	respiratory	France	2005
KT318115	BCoV/FRA/EPI/Caen/2003/05	bovine	enteric	France	2003
KF169915	DEN/03-3	bovine	enteric	Denmark	2003
KF169913	DEN/03-1	bovine	enteric	Denmark	2003
KF169908	SWE/C/92	bovine	enteric	Sweden	1992
M80844	Giessen	bovine	respiratory	Germany	1989
D00731.1	Bovine	bovine	enteric	France	1979
AF391541	BCoV-ENT	bovine	enteric	USA Texas	1998
DQ915164	Alpaca	bovine	enteric	USA Oregon	1998
AF391542	BCoV-LUN	bovine	respiratory	USA Texas	1998
AF058943	LSU-94LSS-051-2	bovine	respiratory	USA Louisiana	1994
AF058944	OK-0514-3	bovine	respiratory	USA	1996
AF058942	LY138	bovine	enteric	USA Utah	1965
AF220295	Quebec	bovine	_	Quebec	1972
U00735	Mebus	bovine	enteric	USA	1972
KF272919.1	RVLC7	bovine	enteric	Ireland	2011

Supplementary Table 2. Details of the strains used for N gene sequences analyses

Accession number	Strain/ isolate	Host species	Type of sample	Country	Sampling year
KU886219.1	BCV-AKS-01	Bovine	-	China	2015
M36656.1	F15	Bovine	enteric	France	1979
EU019216.1	Bubalus/Italy/179/07-11	Bubalus	enteric	Italy	2006
KM985634.1	HLJ-14/CHN	Bovine	enteric	China	2014
EU401983.1	A3	Bovine	enteric	South Korea	1994
EU401984.1	SUN5	Bovine	enteric	South Korea	1994
AB354579.1	Kakegawa	Bovine	enteric	Japan	1976
U00735.2	Mebus	Bovine	enteric	USA	1972
KT318083.1	BCoV/FRA/EPI/Caen/2005/02	Bovine	enteric	France	2005
KT318087.1	BCoV/FRA/EPI/Caen/2003/05	Bovine	enteric	France	2003
KT318084.1	BCoV/FRA/EPI/Caen/2005/01	Bovine	respiratory	France	2005
KT318085.1	BCoV/FRA/EPI/Caen/2007/03	Bovine	enteric	France	2007
KT318096.1	BCoV/FRA/EPI/Caen/2004/14	Bovine	enteric	France	2004
KT318088.1	BCoV/FRA/EPI/Caen/2010/06	Bovine	enteric	France	2010
KT318086.1	BCoV/FRA/EPI/Caen/2008/04	Bovine	enteric	France	2008
MG757140	BCoV/France/11-ICSA16-LBA/2014	Bovine	respiratory	France	2014
MG757141	BCoV/France/ICSA17_LBA/2014	Bovine	respiratory	France	2014
MG757142	BCoV/France/19-pool-LBA/2014	Bovine	respiratory	France	2014
MG757139	BCoV/France/12-ICSA16-EN/2014	Bovine	respiratory	France	2014
MG757138	BCoV/France/ICSA21L3LBA/2014	Bovine	respiratory	France	2014

KT318092.1	BCoV/FRA/EPI/Caen/2013/10	Bovine	enteric	France	2013
KT318089.1	BCoV/FRA/EPI/Caen/2012/07	Bovine	enteric	France	2012
KT318095.1	BCoV/FRA/EPI/Caen/2014/13	Bovine	enteric	France	2014
KX982264.1	BCoV	Bovine	enteric	France	2014
KT318093.1	BCoV/FRA/EPI/Caen/2013/11	Bovine	enteric	France	2013
KT318094.1	BCoV/FRA/EPI/Caen/2014/12	Bovine	enteric	France	2014
KT318090.1	BCoV/FRA/EPI/Caen/2013/08	Bovine	enteric	France	2013
KT318091.1	BCoV/FRA/EPI/Caen/2013/09	Bovine	enteric	France	2013
EU401980.1	0501	Bovine	enteric	South Korea	2005
EU401981.1	0502	Bovine	enteric	South Korea	2005
AF058943.1	LSU-94LSS-051-2	Bovine	respiratory	USA	1994
AF058944.1	OK-0514-3	Bovine	respiratory	USA	1996
FJ425186.1	US/OH-WD358/1994	Waterbuck	enteric	USA	1994
FJ425185.1	US/OH-WD358-GnC/1994	Waterbuck	enteric	USA	1994
DQ811784.2	DB2	Bovine	-	Maryland USA	1984
FJ425189.1	US/OH-WD388/1994	Sambar deer	enteric	USA	1994
EF424621.1	US/OH1/2003	Sable antelope	enteric	USA	2003
EF424623.1	US/OH3/2003	Giraffe	enteric	USA	2003
AF391542.1	BCoV-LUN	Bovine	respiratory	Texas USA	1998
AF391541.1	BCoV-ENT	Bovine	enteric	Texas USA	1998
EF424617.1	R-AH65	Bovine	respiratory	Ohio USA	2001
DQ915164.2	Alpaca	Alpaca	enteric	USA	1998
EF424620.1	R-AH187	Bovine	respiratory	Ohio USA	2000
FJ425187.1	US/OH-WD470/1994	White-tailed deer	enteric	Ohio USA	1994
AF058942.1	LY 138	Bovine	enteric	Utah USA	1965
